# Learning and Generalization under Ambiguity: An fMRI Study

**DOI:** 10.1371/journal.pcbi.1002346

**Published:** 2012-01-19

**Authors:** J. R. Chumbley, G. Flandin, D. R. Bach, J. Daunizeau, E. Fehr, R. J. Dolan, K. J. Friston

**Affiliations:** 1 The Wellcome Trust Centre for Neuroimaging, University College London, London, United Kingdom; 2 Laboratory for Social and Neural Systems Research, Institute of Empirical Research in Economics, University of Zurich, Zurich, Switzerland; University of Oxford, United Kingdom

## Abstract

Adaptive behavior often exploits generalizations from past experience by applying them judiciously in new situations. This requires a means of quantifying the relative importance of prior experience and current information, so they can be balanced optimally. In this study, we ask whether the brain generalizes in an optimal way. Specifically, we used Bayesian learning theory and fMRI to test whether neuronal responses reflect context-sensitive changes in ambiguity or uncertainty about experience-dependent beliefs. We found that the hippocampus expresses clear ambiguity-dependent responses that are associated with an augmented rate of learning. These findings suggest candidate neuronal systems that may be involved in aberrations of generalization, such as over-confidence.

## Author Summary

Intelligent behavior requires flexible responses to new situations, which exploit learned principles or abstractions. When no such principles exist, the imperative is to learn quickly from scratch. Behaviorally, we show that subjects learn action-reward relationships in a manner that enables them to generalize rules to new situations. Our fMRI results show that when subjects have no evidence that such a rule exists, medial temporal lobe responses (that reflect uncertainty) predict their augmented learning.

## Introduction

Successful behavior in new situations often requires us to apply ‘rules-of-thumb’. However, acquiring and applying abstract rules from limited experience presents a fundamental computational problem [1]: in which both over- or under-generalization must be avoided [2], . Despite their importance, little is known about how neuronal systems learn these rules, and how the delicate balance between past and present information is maintained. Evolutionary arguments suggest that the use of previously learned rules when generalizing to new situations increases adaptive fitness by optimizing behavior [7]. This raises the key question of whether and how generalization is optimized [8]. In this work, we examine whether human subjects combine previously learned rules and current information in an optimal way and identify the brain systems that underlie this combination. Using Bayesian learning theory to specify optimal generalization, we looked for its neural correlates. In particular, we drew on existing evidence that points to the hippocampus as a key structure that is implicated in learning the specifics of a new situation, when previously learned rules may not apply [9], [10].

Probabilistic inference in a natural environment is confounded by multiple sources of uncertainty [11], [12], [13], [14], including objective randomness and subjective ignorance [14]. Uncertainty is a key concept here because the confidence about prior beliefs should be weighed against the confidence about new information, when deciding whether to generalize those beliefs to a new situation. Classical reinforcement learning models (e.g. [15], [16]) do not represent uncertainty or use generalization to guide learning and behavior: these schemes simply learn the expected value of action-states and only prosper in environments where the current state is sufficient to specify a successful action: see [17] for a critique and extension. Having said this, several other RL schemes are based on some form of non-probabilistic function approximation and therefore support generalization (see Chapter 8 in [18] for discussion and recent RL approaches in neuroscience that consider generalization in the spatial [19] and temporal [20] case). While recent RL developments in neuroscience incorporate some notion of uncertainty [21], learning and generalization are typically non-probabilistic. In this work we ask if learnt generalizations are accompanied with due uncertainty [22], as prescribed by probability theory.

At the behavioral level, human subjects readily abstract probabilistic rules and use them to generalize [8]. Furthermore, they can distinguish different sources of uncertainty: the unavoidable or irreducible randomness of certain events versus subjective ignorance about the world [12], [13], [23], [24], [25]. The latter resembles the concept of subjective *ambiguity* in economics and represents uncertainty about objective risks. For example, the *risk* (or irreducible randomness) associated with a *fair* coin toss is high (50∶50); however, there may be subjective ambiguity as to whether the coin is itself fair. This paper examines the function and mechanisms of generalization in the face of ambiguity. While there are good reasons to restrict the term ambiguity to *complete* ignorance [26], we use the term more inclusively to denote the level of uncertainty about the outcome probabilities. This is akin to *estimation* [14] or *second-order* [26] uncertainty (i.e., uncertainty about uncertainty). Ambiguity is subjective and reference-dependent: it ranges from complete ignorance to near certainty and, crucially, can be reduced by generalization in a Bayes-optimal fashion [8]. In other words, if subjects consider their current situation in the light of past experience, they can exploit similarities between the past and present to reduce their ambiguity [27], [28]. In our example, ambiguity about a new coin will be reduced by observing the random behavior of similar coins. This ability to generalize over similar situations is seen readily in behavior and learning [8], [14].

In this study, we examined the neuronal correlates of generalization with a special focus on the hippocampus: The hippocampus is involved in generalization [29], [30], [31], [32], [33], [34] and shows activations that are sensitive to objective uncertainty or risk [23], [35]. In this paper, we asked if hippocampal responses also report subjective uncertainty or ambiguity that changes with experience. Specifically, we tested for ambiguity-dependent hippocampal responses, when probabilistic nature of outcomes had to be learned. Furthermore, we hoped to show behaviorally that learning rates were greater in contexts that had more ambiguity. We addressed these questions using a model of our experimental task and, tested whether Bayesian updates or learning could explain behavioral and neurophysiological responses, as measured with fMRI.

## Materials and Methods

### Subjects and procedure

Nineteen subjects (age 19–31, 11 female) were recruited from the UCL psychology Dept subject pool. All subjects gave informed consent, before reading a brief description of the task which was then performed under fMRI. The study protocol was approved by the local UCL ethics committee.

### Image acquisition and analysis

#### Image acquisition

Images were acquired on a 3 T Allegra head scanner (Siemens Medical Systems) with a head coil for RF transmission and signal reception. We used BOLD signal sensitive T2*-weighted transverse single-shot gradient-echo echo-planar imaging (EPI; flip angle 90°; bandwidth BW, 3551 Hz/pixel; phase-encoding (PE) direction, anterior–posterior; bandwidth in PE direction BWPE, 47.3 Hz/pixel; TE, 30 ms; effective TR, 2600 ms). An automatic 3D-shim procedure was performed at the beginning of each experiment. Each volume contained 40 slices of 2-mm thickness (1-mm gap between slices; field of view, 192×192-mm^2^; matrix size, 64×64). Sensitivity losses due to susceptibility artifacts were minimized by applying a *z*-shim gradient moment of 0.4 mT/m, a slice tilt of 30°, and a positive PE gradient polarity [36], [37]. Each subject underwent one scanning session, with three breaks. The task was self-timed, and therefore the duration of each session depended on the subject. The first five volumes of each session were discarded to ensure steady-state longitudinal magnetization.

Whole-brain anatomical scans were acquired using a modified driven equilibrium Fourier transform (MDEFT) sequence with optimized parameters [38]. One hundred seventy-six sagittal partitions were acquired with an image matrix of 256×224 (read×phase) and twofold oversampling in read direction (head/foot direction) to prevent aliasing (isotropic spatial resolution 1-mm;15°; TR/TE/TI, 7.92 ms/2.4 ms/910 ms; BW, 195 Hz/pixel). Spin tagging in the neck was performed to avoid flow artifacts in the vicinity of blood vessels. The flip angle of the tagging pulse was chosen to be 160° to account for B1 losses in the neck. Special RF excitation pulses were used to compensate for B1 inhomogeneity of the transmit coil in superior/inferior and anterior/posterior directions. Images were reconstructed using a standard 3D Fourier Transform, followed by modulus calculation.

#### Image analysis

Functional imaging data were analyzed with statistical parametric mapping (SPM8; Wellcome Trust Centre for Neuroimaging; www.fil.ion.ucl.ac.uk/spm). EPI images were generated off-line using a generalized reconstruction method based on the measured EPI *k*-space trajectory to minimize ghosting. Motion-corrected images were co-registered to the individual's anatomical MDEFT image and spatially normalized to the Montreal Neurological Institute T1 reference brain template (re-sampled voxel size: 2×2×2-mm).

### The experimental paradigm

**Figure 1:**
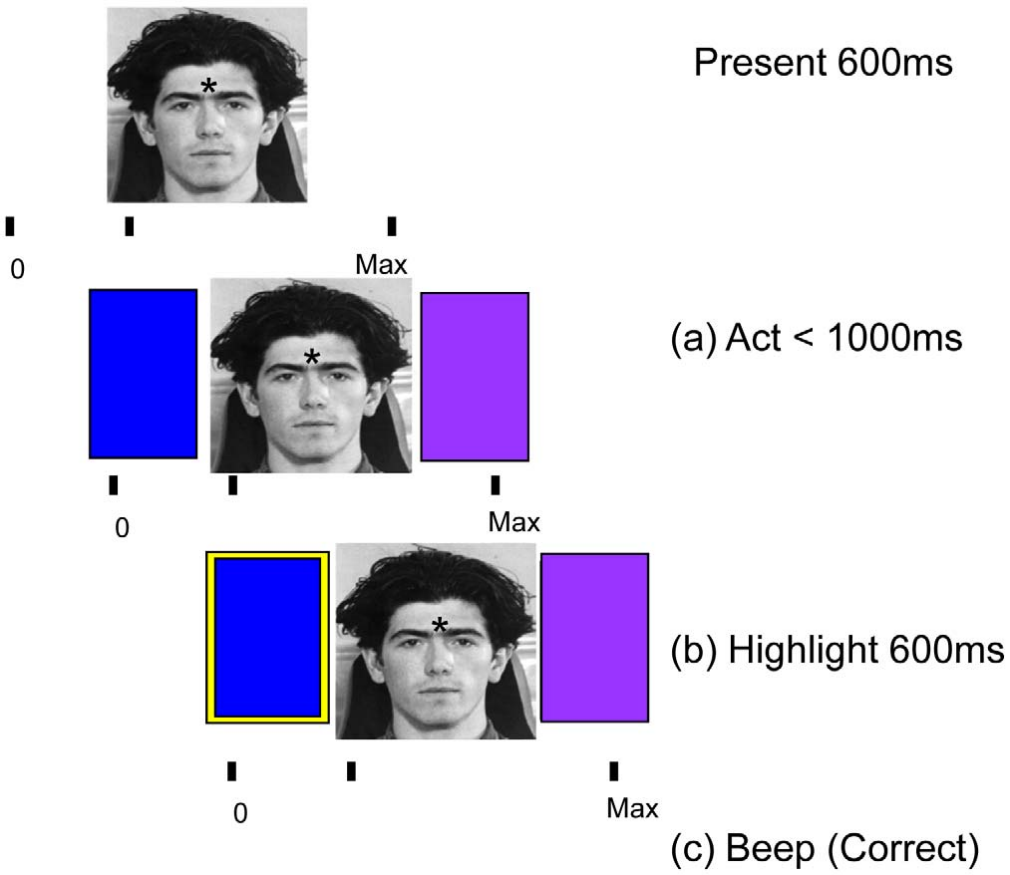
This schematic shows the structure of each trial. A face was presented for 600 ms before two choice options were displayed. The choice options cue the subjects' guess, which was then indicated by a yellow border around the selected option. Audio and visual feedback indicated whether the choice was rewarded (correct) or not (incorrect).

While our goal was to identify domain-general computational processes, the paradigm was framed as a social inference task: Subjects were told that two *groups* of thirty *individuals* had completed a marketing survey. Subjects were then asked to guess, over ten consecutive trials, whether each individual would choose a ‘blue’ or ‘purple’ product. Subjects were told they would be paid ‘in proportion to the number of correct guesses’ and that the two groups were ‘geographically and economically unlike one another’. Trial cues (individuals) were faces from the Sterling data-set, whose group membership was indicated by the symbol ‘*’ or ‘o’ (see Figure 1). Each trial comprised the following sequence: 1) an individual's face was presented along with the symbol indicating their group membership; 2) the response options (blue and purple squares) were then presented, after which 3) the subject responded and 4) received feedback about whether their guess was correct or incorrect. The timeline for a single trial is shown in Figure 1. If subjects did not guess within one second, they were shown the instruction ‘ACT FASTER!’. The subject's guess was highlighted until feedback was delivered. Correct guesses were signaled with an auditory beep (500 milliseconds of 500 Hz sine wave) and accumulated in a score bar at the bottom of the screen. Incorrect guesses were indicated by a 500 millisecond burst of white noise (with no increase in their score).

Unbeknown to subjects, individuals from one group had similar preferences, while the other group had more between-individual variability. This meant that subjects had to make guesses about choices in two distinct contexts established by the group an individual belonged to: in the *generalization context* (GC), all individuals chose ‘purple’ with probability 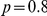. In the *ambiguous context* (AC), ‘blue’ was probabilistically chosen (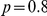) by half of the group members and ‘purple’ (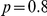) by the other half. To reiterate, subjects were presented with the same face ten times and had to guess whether the individual preferred blue or purple. Each individual was identified as belonging to one group or the other. Every individual preferred one color that was chosen 80% of the time. In the generalization context, all group members preferred the same color, while in the ambiguous context, individual group members preferred blue or purple with equal probability. In both contexts, subjects could learn about any given individual over ten trials.

The generalization context therefore contained a probabilistic rule prescribing the best guess, even in the absence of learning about an individual's preferences. Conversely, in the ambiguous context, subjects had to learn about individual preferences because their group membership provided no clues about what they would preferentially choose. Trials were arranged into blocks, in which the same individual was presented for ten consecutive trials. The blocks alternated between AC and GC, with a new individual (face) for each block. This resulted in 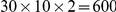 trials, for thirty individuals, presented ten times for two groups.

The blue and purple options were presented with equal probability on the left and right of the screen on each trial. Individuals (faces) were randomly reassigned to either group, between subjects. All subjects experienced the same feedback contingencies (with randomly reassigned cues). Subjects had three short breaks during the task: for each they were first cued ‘PLEASE HAVE A SHORT REST AND RELAX’ before being prompted to restart thirty seconds later: ‘OK! PLEASE PRESS ANY KEY TO CONTINUE’.

### Bayesian modeling versus conventional fMRI analyses

Bayesian learning theory predicts that subjects should learn more quickly about a new individual from the ambiguous group, relative to an individual from the generalization group. This is based upon the assumption that subjects are making Bayes-optimal guesses using a notion of group or context. The increase in learning rate with higher levels of ambiguity is related to increases in learning rate in situations with a high degree of volatility [39] (see below). At the neuronal level, we predicted that increases in learning rate would selectively engage hippocampal processing in the ambiguous context. In other words, hippocampal activation should track changes in ambiguity about an individual's preference as it alternates between AC (high ambiguity) and GC (low ambiguity) blocks. To quantify ambiguity, we assumed subjects were ideal Bayesian observers who used a model of probabilistic outcomes. We focused on two alternative models to predict subject responses, M1 and M2. Under M1, Bayesian learning combines new information with existing generalizations based on group membership. Conversely, M2 accumulates information about every individual independently, without the benefit of generalization.

To make optimal guesses about the choices of each group member, subjects have to infer their preferences i.e. the probability that this individual will choose a particular option, say ‘purple’. We denote this probability with 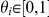. The information following each trial is equivalent to observing the outcome of a biased coin. We use the random variable 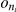 to denote whether the choice of the 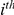 individual was ‘purple’ (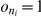) or ‘blue’ (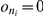): 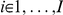 in trial 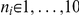 (subjects encountered 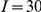 individuals in each of the two groups).

In what follows, we consider alternative models that subjects might have used to infer the 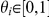. We start with a model that permits generalization and then turn to a version that precludes generalization. We also consider a few alternative models that can be considered as special cases that are of interest from an RL perspective.

### Models

#### M1: Bayes-optimal generalization

The critical feature of *M1* is that guesses about each individual are informed by knowledge about group membership. This model supposes that subjects *jointly* learn about all individuals in a given group. In the generalization context, subjects should be more confident about a new individual from the unambiguous group, relative to the ambiguous group that provides no contextual clues. This differential uncertainty (ambiguity) is our focus. For simplicity, we assumed that subjects generalize within, but not between, groups. In other words, learning in one context was independent of learning in the other. An additional hierarchical level would permit generalization across contexts (e.g., the relative size of each group) and could be modeled with an extension of the Bayesian framework described below [40].

The form of our model appeals to behavioral evidence that human rule learning resembles non-parametric Bayesian inference [41]. It is also related to a previous [42] Bayesian formulation of rule-learning. (While the latter model focuses on Pavlovian learning, it resembles M1 through inferring the hidden number of subgroups or ‘latent causes’ [42]). In our model, subjects represent the (preferences of) individuals, 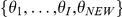 where 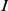 is the total number of individuals encountered so far. Subjects represent individual preferences by assigning individuals to subgroups, according to their similarity. Note that while subjects observe group membership, subgroup membership is hidden: There are two hidden subgroups in the ambiguous context, preferring either blue or purple, but only one in the generalization context. By first finding the number and nature of subgroups, optimal Bayesian assignment avoids over-generalization (e.g. incorrectly labeling a new blue-preferring individual as belonging to a known purple-preferring subgroup) and under-generalizing (e.g. failing to recognize that a new purple-preferring individual belongs to a known purple-preferring subgroup). This type of learning has had considerable success in modeling category learning in humans [41] and ‘rationalizes’ non-Bayesian models of generalization in reinforcement learning [43] (see below).

We assume that subjects store the number of times (out of 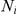) the 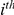 individual chose ‘purple’. The cumulative counts up to the present trial 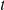, are denoted by 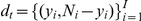 where 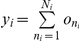. Subjects model their cumulative observations 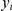 as drawn from a mixture of Binomial distributions of the form 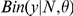. Being ignorant of the mixing distribution 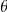, we assume they use a Dirichlet process 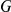 over 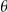, with concentration parameter 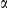 and base distribution 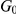 [corresponding to the uninformative conjugate Beta distribution 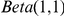]. These define the base measure 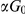. The resulting probabilistic model is
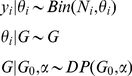
(1)Here 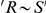 means 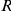 has the distribution 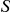; so the right hand side specifies a distribution. The Dirichlet process, DP, is thus a distribution on distributions and models ambiguity. Because realizations of a DP are discrete with probability one, these models can be viewed as probability measures consisting of a weighted sum of point masses [44], [45]; i.e., countably infinite mixtures
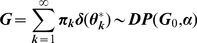
(2)Here 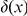 is a point mass at a single point 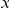, 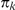 is a stick-breaking process and 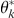 is distributed as 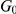 [46].

The implicit form of generalization is more transparent when we integrate over 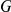 to obtain a prior over 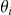 in terms of successive conditional distributions (see [46] for a measure-theoretic proof of this integral)
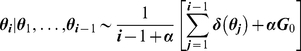
(3)This means the prior belief about one individual 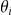 depends on knowledge about others sampled from the population, 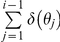, as well as the initial distribution 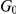. This completes our description of M1 in terms of a likelihood (in Eq. 1) and prior (in Eq. 2/3).

To predict subject's responses we require M1's posterior belief about the behavioral contingencies. This quantifies the ambiguity as well as the value of their response options. For posterior inference, one can obtain a sample from the posterior of 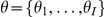 by simulating a Markov chain whose equilibrium distribution is the desired posterior distribution [47]. The simplest approach is to repeatedly sample 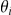 from its conditional distribution, given both the data and all other 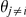, denoted by 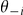. This distribution therefore combines the likelihood of 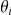 and the prior, conditional on 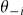. This conditional prior for an individual based on previous individuals is given by
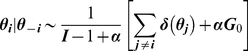
(4)and derives from the previous equation by noting that 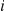 is the last of 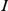 observations (i.e. by assuming the 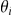 are exchangeable). Introducing the likelihood, this yields the following conditional posterior distribution: 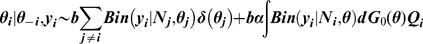
(5)Where 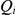 is the posterior over 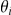, based on the prior 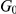 and the single observation 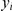 with likelihood 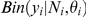, *i.e.* 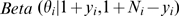. Here, 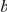 is chosen to ensure that
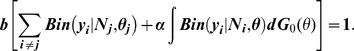
This Bayesian model is related to the non-Bayesian RL model of [43] mentioned in the introduction. In that RL model, each cue is first ‘classified’ before reinforcement learning. A cue is either assigned to a known class of cues based on similarity, or designated exceptional and given its own class. Both perceptual similarity and predictive similarity play a role: do two cues look the same? do they predict the same outcomes? Regarding the latter, *negative prediction errors* from RL reduce perceived similarity between cues in a separate *recognition system*, thereby promoting discrimination over generalization [43]. Our focus is on this predictive similarity. To derive *optimal* generalization, we define predictive similarity as the likelihood of an outcome, given a cue (rather than the inverse magnitude of a negative prediction error). In particular, a cue's past associations determine if it will be assigned to a known class based on similarity, defined by 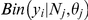, or assigned to its own class with probability 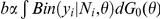. The hyperparameter 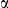 controls this tradeoff between generalization and discrimination and can itself be learned [48].

Having assumed 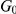 is an uninformative Beta distribution, 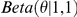, which is conjugate to the likelihood, calculating the integral 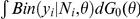 and sampling from 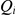 are straight-forward. The simplest algorithm [47] for Gibbs sampling from the full posterior 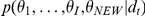 including 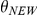 is (see [47], [48] for further details):

*For* 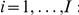


→*Draw a new value from* 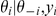 *as defined above.*

*With probability* 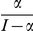 *draw* 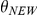 *from the base distribution* 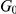.

→*Otherwise, uniformly draw one existing* 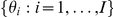 *and assign its value to* 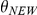.

This procedure approximates the trial-by-trial evolution of posterior belief about preferences, 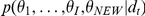. Once the Markov chain has reached equilibrium, we use a sample of size 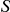 from that distribution. Furthermore, any marginal posterior of interest 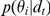 or 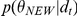 is approximated simply as the univariate component of this joint sample [49].

We used two measures of this time-dependent posterior as explanatory variables to predict the behavioral and neurophysiological responses of each subject. Firstly, we operationalized the ambiguity about each new individual using the Shannon entropy 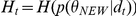. To evaluate this entropy, univariate samples from 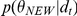 were first binned into 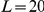 bins to provide an approximate discrete probability mass function with 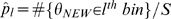. In this case, we have 
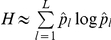
. This uncertainty measure characterizes the ambiguity about a new individual, as generalized from experience with other individuals in the group. A differential entropy between AC/GC reflects both the number of subgroups (clusters) and uncertainty about the parameters describing those subgroups. To see this more clearly take an extreme case where all 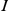 individuals encountered so far have been attributed to one subgroup, 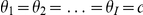. This subgroup, characterized by a parameter setting of 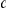, is therefore strongly favored as an *a priori* explanation for new individuals (i.e. strong generalization). Applying this condition to Eq. 3 gives
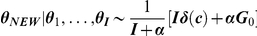
Intuitively, 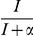 probability mass now rests on just one point mass (for small 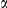 this is all the mass). This predictive distribution is therefore less ‘dispersed’ than if there were two or more subgroups (i.e. it has lower entropy). For this reason, when 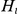 is small, learning is more strongly biased towards belief in one subgroup. In this sense, the entropy can be regarded as a proxy for ambiguity that dictates the ‘learning rate’ or the sensitivity to new information. We used this entropy measure to identify the neurophysiological correlates of ambiguity, using fMRI responses.

We have emphasized that greater prior ambiguity (i.e. higher number of inferred subgroups/higher predictive entropy) is accompanied by a diminished *a priori* bias. This affords observations more influence over posterior belief. Another influential hypothesis is that uncertainty influences choice by promoting exploration itself [50], [51] i.e. what to learn about *vs.* how fast to learn in the current situation. To simplify things, we chose a task with no exploration-exploitation trade-off. Specifically, because every trial in our task provides feedback on the value of the chosen action *and* counterfactual information about the other unchosen action, there is no information to be gained from exploring the less valuable action.

Subjects do not know the true expected reward, 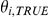, for choosing ‘purple’ when faced with the 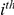 individual (they do not know that individual's preference). Let 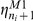 then denote their subjective, expected reward for guessing ‘purple’ on the 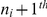 trial faced with the 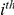 individual, following a total of 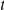 trials under M1. This expectation is defined by weighting possible values of 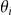 according to their current plausibility, giving 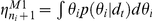. This is just the posterior expectation of 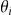 and can be approximated by
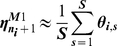
(6)Here, each 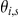 is an MCMC sample from the posterior 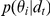 conditional on all observations to date. This replaces an analytic expectation with an empirical expectation (converging according to the law of large numbers). An exactly analogous approximation yields the predicted value for a new cue:
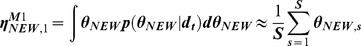
(7)We now turn to some alternative models.

#### M2: Rescorla-Wagner without generalization

To assess the predictions of M1 in relation to a null model, we also considered the predictions under M2, where subjects learn about each individual without generalization. Under this assumption, the expected reward (correct choice) can be modeled with classical Rescorla-Wagner learning [15].
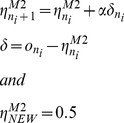
(8)Where 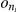 is still the binary outcome on trial 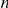 with the 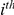 cue. One implementation of M2 - akin to habit learning – would be to separately initialize the value of guessing ‘purple’ or ‘blue’ (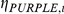, 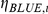) to zero and update each only when the corresponding action was taken [52]. Guesses could then be modeled according to 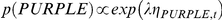, where 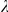 controls the stochastic precision of the guess. However, because subjects are told that exactly one option is correct, each outcome is informative about the counterfactual (unchosen) option. We therefore initialized 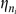 (the value of the purple guess on trial 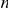 in the presence of each cue 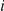) to 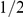, and defined the value of the blue choice as 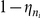. Subsequent outcomes 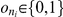 push 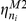 up or down as specified by M2. This agent therefore uses counterfactual data (from the unchosen option), but does not generalize between individuals. We fit the free ‘learning rate’ parameter 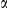 by minimizing the error function, 
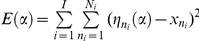
, where 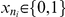 indicates which option the subject guessed on the corresponding trial (coded as 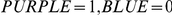). 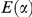 was evaluated numerically for different values of 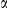 with increments of 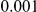 within parameter space.

#### M3–M5: Additional models

**Figure 2:**
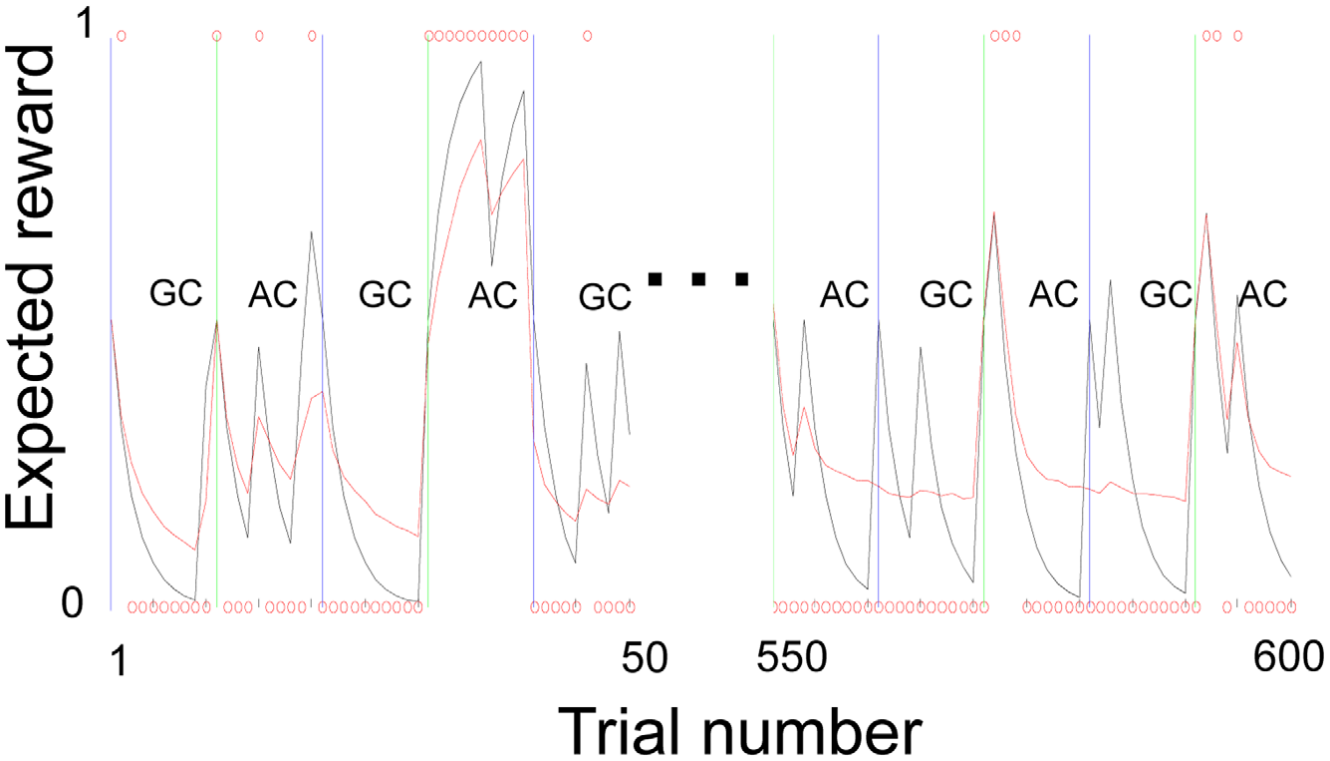
Subject's predicted value (expected reward) for guessing ‘purple’, according to model-based (M1, red-dashed) and model-free (M2, black) schemes. M1 tracks current information when necessary (AC), and otherwise exploits generalization to limit the impact of spurious outcomes on action (GC). M2 is ignorant about each new individual and myopically chases reward. Red circles indicate the actual guesses of a typical subject.

**Figure 3:**
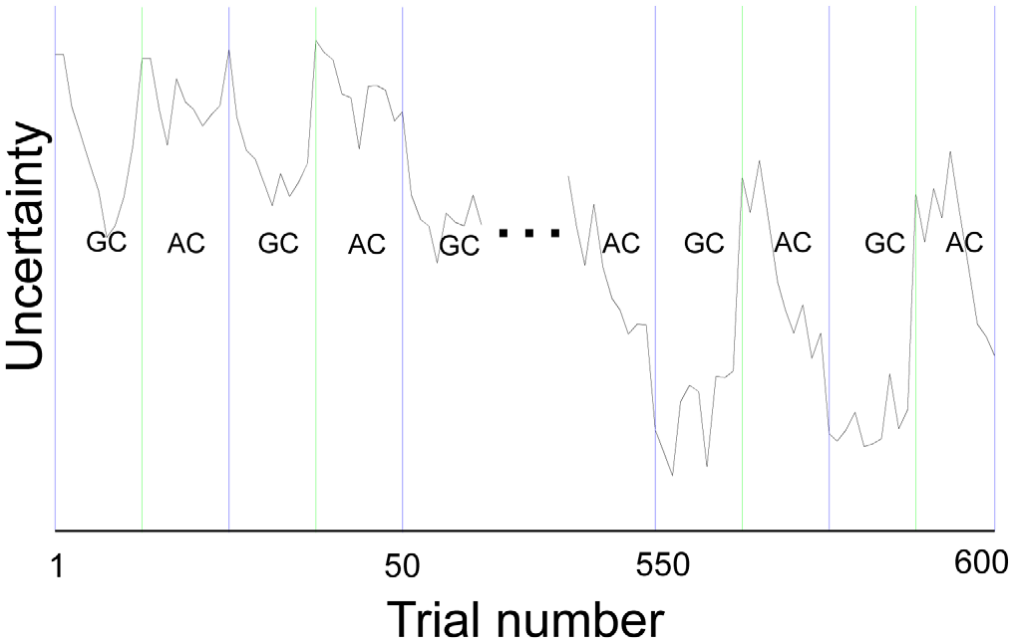
Degree of learning formalized as prior uncertainty about the reinforcement contingencies, in M1. Without evidence of a contextual norm (in the AC) subjects are uncertain about what to do with an unfamiliar person, and must learn quickly. This time-series, convolved with a hemodynamic response function predicted hippocampal fMRI responses (see main text).

The resulting sequence of value for our two models 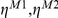 are plotted as a function of trial number in the dashed and solid curves of Figure 2, for the sequence of face cues and outcomes presented to our subjects (i.e. the sequence of blue/purple choices made by each individual). These correspond to the value of guessing purple under a model with (M1) and without (M2) the facility for generalization. For ease of visualization, Figure 2 and 3 interpolate discrete-time model predictions to form smooth curves. The vertical lines demarking the context (ambiguous or generalization) are centered on the first trial of each block.

To ensure we had not overlooked other explanations for the subjects' responses, we performed secondary analyses, to establish the explanatory power of M1 in the context of alternative models, M3–M5. M3 was a generalization of M2 [15], which represents and learns the value of contextual (group-membership) cues. This agent therefore represents 62 cues (60 faces, 2 contextual cues). On each trial, M3 calls and updates both the context (group) *and* individual (face) cues presented on that trial. Defining 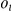 as the outcome on trial 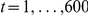 and 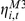 as the instrumental value of choosing purple, faced with the 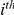 cue, updates were implemented with
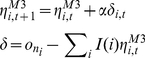
(9)where the indicator 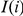 is 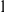 whenever the 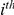 cue is present and 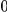 otherwise. Only values for cues actually present in trial 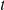 are updated. Like M2, this agent uses counterfactual information (from the unchosen option). Specifically, the value of choosing ‘purple’ on trial 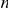 was 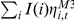 and the value of choosing ‘blue’ was 
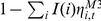
. Each 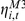 was initialized to 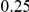 so that prior to learning ‘blue’ or ‘purple’ were equally valuable; i.e., 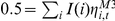. The free parameter 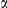 was fit to each subject's guesses using the same procedure as for M2. M4 modeled a Bayesian learner that *over-generalizes*. It has an identical mathematical form to M1 but unlike M1 does not distinguish between contexts. It treats all individuals from the two contexts/populations indiscriminately i.e. as part of one ‘meta-population’. M5 modeled a Bayesian learner that *under-generalizes*; In other words, it can represent uncertainty but cannot generalize. M5, like M1 and M4, models observations associated with any one individual as 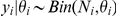, but differs in the prior. In particular, each individual is treated independently with no generalization within or between groups (the prior over individuals factorizes). Specifically, we use a Beta prior that resets the prior for the 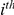 cue to uniform 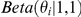, irrespective of its experience with other cues. This agent shares a key feature of M2 - resetting the predictions for each new cue to *0.5* and learning without generalization. For subsequent trials, it calculates the expected value of choosing purple as
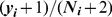
(10)where 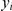 is still the count of correct purple choices with cue 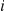 (see above and [49]). In practice this agent's predictions are similar to M2.

### Relating model predictions to data

#### Behavior

We used logistic regression to predict trial-by-trial choices from the value (expected reward) based on 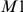, while including the value derived from models 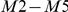 as additional nuisance covariates. We calculated within-subject point estimates of the partial regression coefficients of 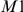 predictions, before testing for significant (nonzero) effects at the between-subject level using standard classical statistics.

#### fMRI

Our analyses of the fMRI data used a conventional approach in which the parametric effects of variables from our formal Bayesian model were used to predict the amplitude of fMRI responses, after convolution with a suitable hemodynamic response function [53]. Because the majority of experimental variation in the model predictions is between conditions (AC vs. GC), we arranged these conditions in a block design to ensure high efficiency. We could therefore choose either a conventional analysis that simply tested for condition effects or a model-based analysis that used parametric variations within and between conditions. To exploit our formal model, we used the more comprehensive model-based analysis: The fMRI data were modeled using a general linear convolution model, whose explanatory variables comprised stimulus functions convolved with a canonical hemodynamic response function. These stimulus functions comprised delta functions modulated by the following:

1) The prediction ‘risk’ under M1 (time-locked to the choice presentation): 2) The reward predictions under M1, conditioned on the subject's choice (time-locked to the choice): 3) The model-based (Shannon) surprise at the outcome under M1 (time-locked to the outcome): 4) The signed model-based prediction error under M1, conditional on subject's choice (time-locked to the outcome): 5) the trial outcome: correct/incorrect, coded at 1,0 respectively (time-locked to the outcome). In terms of our hypothesis, these regressors can be regarded as modeling nuisance effects. Our final regressor was the key effect of interest; namely, the trial specific ambiguity as measured by the Shannon entropy above. It is this measure that reflects an encoding of contextual uncertainty that weakens generalization. The entropy entered as parametrically modulated delta functions at the time of choice, but before feedback. Six columns describing scan-specific rigid body translations and rotations were included as confounds. The data was temporally filtered to remove low-frequency drifts below 1/128 Hz.

## Results

Figure 2 shows the value (expected reward) of each choice according to the two main learning models we considered, together with a typical subject's guesses. Model 1 (M1) generalizes, while Model 2 (M2) cannot. For each subject, we used logistic regression to explain their choices in terms of these predictions and a constant term. Using a between-subject summary-statistic approach, we applied a two-tailed Student's *t*-test to the subject-specific logistic regression coefficients associated with the predictions of M1 (red-dashed curve, Figure 2). We rejected the null hypothesis that this effect was equal to zero (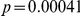, 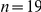). Interestingly, the size of the M1 regression coefficient predicted the total number of rewards obtained by each subject (correlation 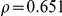, 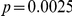 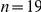). This illustrates that generalization is evident behaviorally and pays off.

A secondary behavioral analysis assessed the specificity of M1 predictions by examining the explanatory power of M1 in the context of the alternative models, M2 to M5. For each subject, we used logistic regression to explain subject's choices as a mixture of predictions from five models (M1 to M5), plus a constant term. Having estimated the logistic regression model for each subject, we again considered the subject-specific estimates for the coefficient reporting on M1 predictions. A two-tailed Student's *t*-test on the M1 coefficients was highly significant, 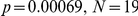. No other model coefficients reached significance.

To summarize, we used standard regression techniques to ask if, having accounted for competing models, a component of choice behavior reflects Bayes-optimal generalization (M1). Specifically, we included several model predictions in one linear model and estimated the partial regression coefficient for the predictor of interest (action-values derived from M1). One can therefore [54] conclude that, over and above competing models, behavior can be predicted by M1. Because Models M2 and M3 have a free parameter this conclusion is conservative: having been pre-fit to subject's behavior, these models have an explanatory advantage that is unavailable to M1 (or M4 and M5). In contrast to M2 (Eq. 8), M1 attempts to explain behavior via abstract computational principles, not detailed mechanisms. Its predictions have no free parameters. Rather, its predictions are based only on the subject's observations under ideal Bayesian assumptions. We have demonstrated that this model predicts behavior, above and beyond that explained by the other models considered. In what follows, we now ask whether the brain encodes ambiguity [see e.g. [39] for a similar approach].

**Figure 4:**
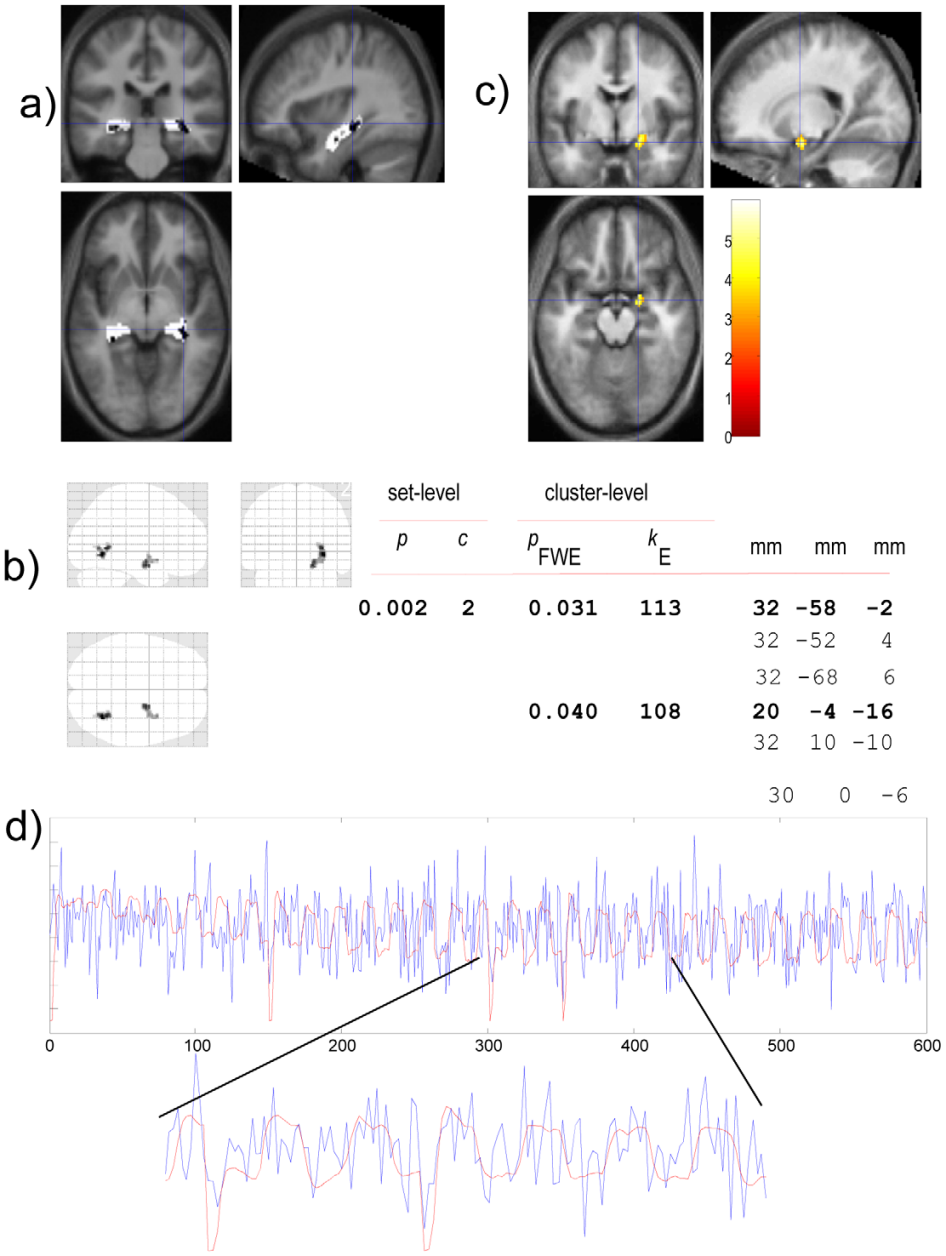
Structural MRI, mask and functional activation. (**a**) Structural region-of-interest (white) on the subjects' average anatomical image. For visualization, black blobs exceed the 0.05 level uncorrected threshold corresponding to a Student's *t* (13 df). See the main text for statistical inference at a corrected p<0.05 level. (**b,c**) Whole brain analysis of the effect of ambiguity. (**b**) Shows a glass-brain view (maximum intensity projection) of significant activations. (**c**) Shows the anterior activation, which included right anterior hippocampus and amygdala as defined, superimposed on the subjects' average anatomical image. (**d**) The observed fMRI trial-by-trial time-series (blue) averaged over all subjects for the hippocampal activation identified in our whole brain analysis (see main text). The model-based ambiguity is shown in red. Note that the model only captures the slow changes in observed responses over blocks as the contingencies are learned.

While M1 differs from other models in many ways, the important aspect for the fMRI analysis is that M1 provides an ambiguity measure. We therefore tested the null hypothesis that the fMRI signal is sensitive to ambiguity, as quantified by the Shannon entropy of prior belief (see above). In our fMRI data, fourteen subjects satisfied the inclusion criteria for a second-level between subject analysis (no interruptions to the scanner session or rapid head movement, as estimated by co-registration). We conducted regional and whole-brain analyses. All fMRI results presented here are based on the same general linear model, including the confounding factors (i.e., with nine regressors). In view of our specific hypothesis, region of interest (ROI) analyses asked whether activity within bilateral hippocampi tracked ambiguity about the current contingencies. Figure 4a shows the anatomy of the ROI.

Figure 3 depicts ambiguity about a new individual (alternating block-wise between GC and AC blocks). As discussed, this dictates the relative influence of the current observation on belief updates (higher when there is high ambiguity). The parameter estimates associated with the entropy regressor above were averaged over bilateral hippocampal voxels for each subject, using the AAL atlas [55]. We applied a two-tailed Student's *t*-test to these subject-specific summaries, testing the null hypothesis that hippocampal responses do not covary with ambiguity. We were able to reject this null hypothesis with a correct 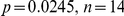. Repeating the analysis on unilateral right and left hippocampus separately provided similar results (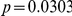, 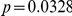, respectively). (These latter two results examine the separate contribution of each hemisphere to our bi-lateral effect. These tests are not statistically independent of the bi-lateral test and were not subject to additional correction.) There was no significant difference between left and right hippocampi. Our results therefore suggest that neuronal activity encodes the same sorts of variables that arise in our Bayes-optimal computations and, consequently, may be performing some form of approximate Bayesian inference.

As with the behavioral data, we next examined the between-subject correlation between the hippocampal ambiguity coefficients and the total number of rewards attained in the experiment (correlation 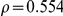, 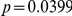 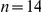). Testing for separate correlations in left and right hippocampal effects gave respectively: 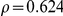, 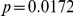 and 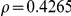, 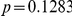 (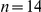).

In an exploratory whole brain analysis, we then smoothed the data with a Gaussian Kernel 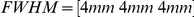 and re-estimated the general linear model above using a conventional SPM analysis with whole brain correction for multiple comparisons [56]. Two right-hemisphere clusters survived correction for cluster-extent (using a height threshold of 3). The first region (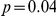 FWE corrected) subsumed a right hippocampal region, mostly hippocampus and amygdala, but also putamen, as defined with the AAL atlas [55]. The second region (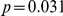 FWE corrected) encompassed the fusiform gyrus and precuneus, with a spill-over into a calcarine region. These regions are shown in maximum intensity projection format in Figure 4b (this display format shows voxels with maximum intensity that fall on parallel lines traced from the viewpoint to the plane of projection as in a standard X-Ray). Orthogonal views of the anterior activation at its local maximum are shown in Figure 4c. For illustration purposes, Figure 4d shows the mean times series in this anterior region, averaged over all subjects. All of the above fMRI analyses were based on the same model, which included the nuisance regressors listed in **Relating model predictions to data: fMRI**. None of these nuisance effects could explain the variation in hippocampal responses that was explained by our Bayes-optimal generalization model (M1).

## Discussion

Behaviorally, we have shown that subjects learn action-reward relationships in a manner that enables them to generalize rules to new situations. Crucially, this enables subjects to adapt their learning rate to provide an optimal balance between pre-existing generalizations and new information. We established this by showing that the accuracy of subjects' guesses evolved over trials in a way that was predicted by Bayes-optimal generalization, using a statistical model equipped with prior beliefs that allowed for contextual ambiguity. Furthermore, we established that a significant component of hippocampal responses could be explained by fluctuations in ambiguity under this model. These regionally specific responses were also significant in a whole brain SPM analysis.

We provide empirical support for a model that explains how experience moderates decision making. In this model, the bias towards rule-based choices is determined by low ambiguity. We show that both learning and hippocampal responses are attenuated when the underlying rule is learned and applied in an unambiguous context. Conventional ‘model-free’ reinforcement learning cannot easily explain such effects because these schemes do not include contextual ambiguity. As noted in the introduction, one recent variant of reinforcement learning [43] is relevant here: In this two-system learning theory, generalization between observable cues rests both on their perceptual similarity and their predictive similarity (do cues look the same? do they predict the same outcomes?). The authors of [43] contrast normal learning with under/over-generalization or ‘under/over willingness to generate a new state’ p 97. We have used a single model that formalizes this optimality by drawing on principles of optimal probabilistic generalization (see [42] for a related model). As in [43], our model generalizes by classifying observable cues before acting. Unlike [43], it invokes an explicit representation of subjective ambiguity to mediate and optimize this generalization. There remains an interesting challenge to relate our formulation and results to classical RL schemes. Interestingly the authors of [43] speculate that the neuronal systems mediating generalization depend on the hippocampus (and PFC); because these systems are flexible, the rules by which observable cues are classified can easily be changed to permit new discriminations. These speculations are entirely consistent with our findings.

As in previous treatments [14], we distinguish uncertainty about objective, observable events (e.g., the risk of getting ‘tails’ in a fair coin flip) from subjective ambiguity about unobservable states or parameters (is the coin really fair?). While the hippocampus has been implicated in the former [23], [35], [57], the latter is central to computational accounts of contextual learning and inference; e.g. [1], [22]. Using a Bayes-optimal model, our work provides the first evidence that the hippocampus tracks contextual ambiguity about hidden or latent variables.

Previous work [11], [14], [39], [58] has addressed how ambiguity mediates the influence of uncued *temporal* variability (volatility) on learning. We asked if variability in response requirements to different cues influences creates ambiguity and influences learning. In the current study, we manipulated the uncertainty about the behavioral contingencies over contexts, rather than time, and showed that associative learning adapts accordingly. Further work could examine whether neuromodulatory manipulations influence this effect; e.g., by selectively facilitating synaptic gain as predicted by [11], [59]. The role of dopamine deserves special attention, given prior work with Pavlovian or simpler instrumental tasks [60]. Additionally, given that the amygdala is able to modulate memory storage in non-amygdala brain areas [61], multi-region *in vivo* recordings could disclose interactions with the hippocampus in these tasks. Interestingly, the amygdala activation in our whole-brain analyses is consistent with previous work implicating the amygdala in the representation of ambiguity [24], [62]. However, previous studies were unable to address whether ambiguity regulates learning, as predicted theoretically. In line with Bayesian learning theory, our results suggest that learning (updating beliefs) can be guided by optimal probabilistic constraints, generalized from previous experience.

The learning rate in (model-free) reinforcement learning prescribes the sensitivity of belief updates to current information. When this information is under or over-weighted, inefficient learning ensues. While classical RL is non-probabilistic (i.e. has a degraded uncertainty representation [22]), it may in principle address this challenge by incorporating something akin to an ‘ambiguity-dependent’ or ‘surprise-dependent’ learning rate. For example, attempts have been made to optimise learning rates [63], [64] in both stationary and non-stationary settings [65]. Bayesian learners use the rules of probability to achieve this balance by weighing new information against pre-existing generalizations. The relative weight of the latter depends upon ambiguity (the relative confidence in prior beliefs about the current context). When pre-existing beliefs are held with a high degree of confidence, they generally accommodate new observations, by down-weighting their impact. Such abilities to balance different sources of information and constraints are at the heart of adaptive behavior [66]. For example, appropriate social behavior requires communal norms, while retaining sensitivity to individual inclinations and preferences. The (social) learning task in this paper is a first step in this direction. Conversely, aberrant generalization has widespread consequences [2], [3], [5], [67]. The framework used in this study may provide an experimental framework to quantify dysfunctional generalization in specific patients; e.g., over-generalized schemata which persist despite contradictory evidence, as seen in depressive and delusional states and its associated pathophysiology at the neuronal level.
